# Adrenomedullin in Ovarian Cancer: Foe In Vitro and Friend In Vivo?

**DOI:** 10.1371/journal.pone.0040678

**Published:** 2012-07-30

**Authors:** Cinzia Baranello, Marisa Mariani, Mirko Andreoli, Mara Fanelli, Enrica Martinelli, Gabriella Ferrandina, Giovanni Scambia, Shohreh Shahabi, Cristiano Ferlini

**Affiliations:** 1 Department of Oncology, Catholic University of the Sacred Heart, Campobasso, Italy; 2 Danbury Hospital Research Institute, Danbury, Connecticut, United States of America; University of Campinas, Brazil

## Abstract

Stromal elements within a tumor interact with cancer cells to create a microenvironment that supports tumor growth and survival. Adrenomedullin (ADM) is an autocrine/paracrine factor produced by both stromal cells and cancer cells to create such a microenvironment. During differentiation of macrophages, ADM is produced in response to pro-inflammatory stimuli and hypoxia. In this study we investigated the role of ADM as a growth factor for ovarian cancer cells and as a modulator of macrophages. We also analyzed ADM expression levels in a retrospective clinical study using nanofluidic technology and assessment of ADM at the gene level in 220 ovarian cancer patients. To study the effects of ADM, ovarian cancer cell lines A2780, OVCAR-3, and HEY and their drug-resistant counterparts were used for proliferation assays, while monocytes from healthy donors were differentiated in vitro. ADM was a weak growth factor, as revealed by proliferation assays and cell cycle analysis. After culturing cancer cells under stressing conditions, such as serum starvation and/or hypoxia, ADM was found to be a survival factor in HEY but not in other cell lines. In macrophages, ADM showed activity on proliferation/differentiation, primarily in type 2 macrophages (M2). Unexpectedly, the clinical study revealed that high expression of ADM was linked to positive outcome and to cancer with low Ca125. In conclusion, although in vitro ADM was a potential factor in biological aggressiveness, this possibility was not confirmed in patients. Therefore, use of an ADM antagonist would be inappropriate in managing ovarian cancer patients.

## Introduction

Cancer microenvironment is a topic that has recently begun to interest investigators. For years research focused on the cancer cell itself, but a solid tumor also contains nonmalignant stromal elements, including macrophages, lymphocytes, mast cells, endothelial cells, fibroblasts, myofibroblasts, pericytes, and mesenchymal stem cells, all of which interact with cancer cells to create a microenvironment that supports tumor growth and survival [1], [2]. Understanding the exact composition of the tumor microenvironment could be useful for the development of rational therapeutic approaches.

Epithelial ovarian cancer exhibits a complex cytokine–chemokine network [3]. Receptors for chemokines are expressed in a variety of infiltrating cells, including macrophages, which are recruited by chemokines [4]. This interaction is a 2-way process: ovarian cancer cells are also capable of modulating the macrophage phenotype, since the cytokines and cell surface receptors which are induced in co-cultured macrophages in vitro are also detected in human ovarian cancer [5]. ADM is capable of promoting angiogenesis and melanoma growth [6] both via the paracrine effect, mediated by the endothelial nitric oxide synthase signaling pathway, and by the autocrine effect, which stimulates the polarization of macrophages toward an alternatively activated phenotype (M2).

ADM is a 52–amino-acid soluble peptide that was purified for the first time from phaeochromocytoma [7] and thought to be mainly produced by the adrenal medulla. Today, however, it is well known that ADM is nearly ubiquitous, with widespread tissue distribution that is indicative of its multiple biological activities [8]. ADM is produced by both stromal (ie, endothelial, vascular smooth muscle, myocardial, and central nervous system) and tumor cells as an autocrine/paracrine factor. It is also produced during differentiation of macrophages in response to pro-inflammatory stimuli and hypoxia, and it has mainly anti-inflammatory effects, since ADM inhibits TNF-α production by activated macrophages, reduces vascular permeability, and downregulates Th1-mediated autoimmune response [9].

**Figure 1:**
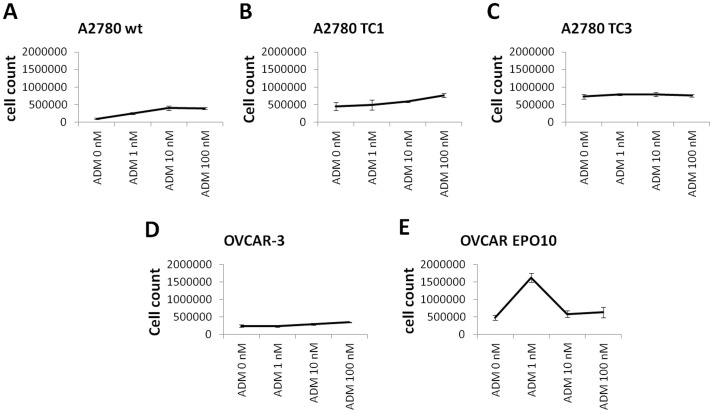
Effect of ADM on cell growth. A2780, TC1, TC3, OVCAR-3, and OVCAR-EPO10 cells were treated with ADM 1, 10, and 100 nM for 72 hours and counted. Cell counts are shown as absolute number of cells. A modest effect on cell growth was noticed in some cell lines, such as A2780 (A), TC1 (B), and OVCAR-3 (D) cells.

Structurally, ADM is characterized by a typical 6–amino-acid ring, due to a disulfide bridge between cysteines 16 and 21, and it is amidated at the C-terminus. Both the disulfide bond and amidation are essential for its activity. ADM is homologous to calcitonin gene-related peptide (CGRP) and amylin, both members of the calcitonin/CGRP/amylin superfamily [10]. This homology allows significant cross-reactivity with the receptors of these other peptides, as well as with calcitonin itself. Two receptors have been identified for ADM: ADM-R1 and ADM-R2, both formed by a common main subunit and an accessory protein. The main subunit is the calcitonin receptor like receptor (CRLR); the accessory protein is the receptor activity-modifying protein 2 (RAMP2) in ADM-R1 and RAMP3 in ADM-R2 [11].

Since ovarian cancer is a disease that spreads in an environment rich in macrophages, which are believed to be the main source of ADM production, in this study we investigated the role of ADM as a growth factor for cancer cells and as a modulating factor in macrophages. Although in vitro we noticed its partial activity as a factor promoting growth in cancer cells, in a retrospective clinical study we observed that high levels of ADM are linked to a positive outcome and to a cancer with low Ca125 expression.

**Figure 2:**
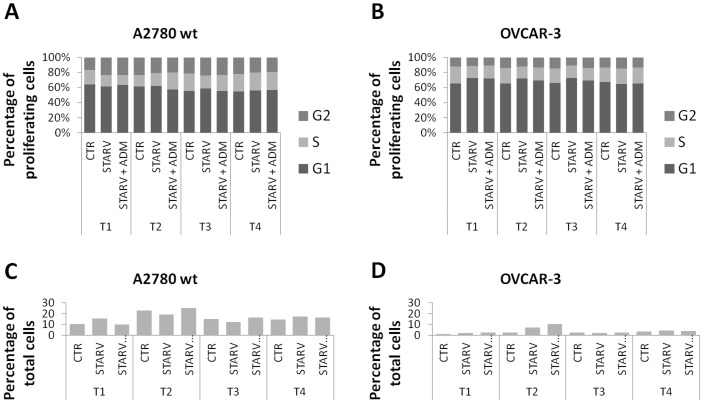
Effect of ADM on cell cycle in A2780 and OVCAR cells. Cells were starved for 36 hours with or without addition of ADM 100 nM, harvested after 0 (T1), 4 (T2), 8 (T3), and 24 hrs (T4), fixed, stained with propidium iodide and acquired using a cytofluorimeter. Data were analyzed as histogram plots. By plotting cell count versus propidium iodide (PI) fluorescence, the percentage of cells in each phase was evaluated. Data from A2780 and OVCAR-3 cells alone are shown as a percentage of the total G1+S+G2 (A–B). Cell death was also evaluated by analyzing the DNA fragmentation included in the sub-G_0/1_ peak (C–D). The effect of ADM on cell cycle and on cell death is not detectable. This experiment was repeated twice with similar results.

## Results

### ADM is a Weak Growth Factor for Ovarian Cancer Cell Lines

**Figure 3:**
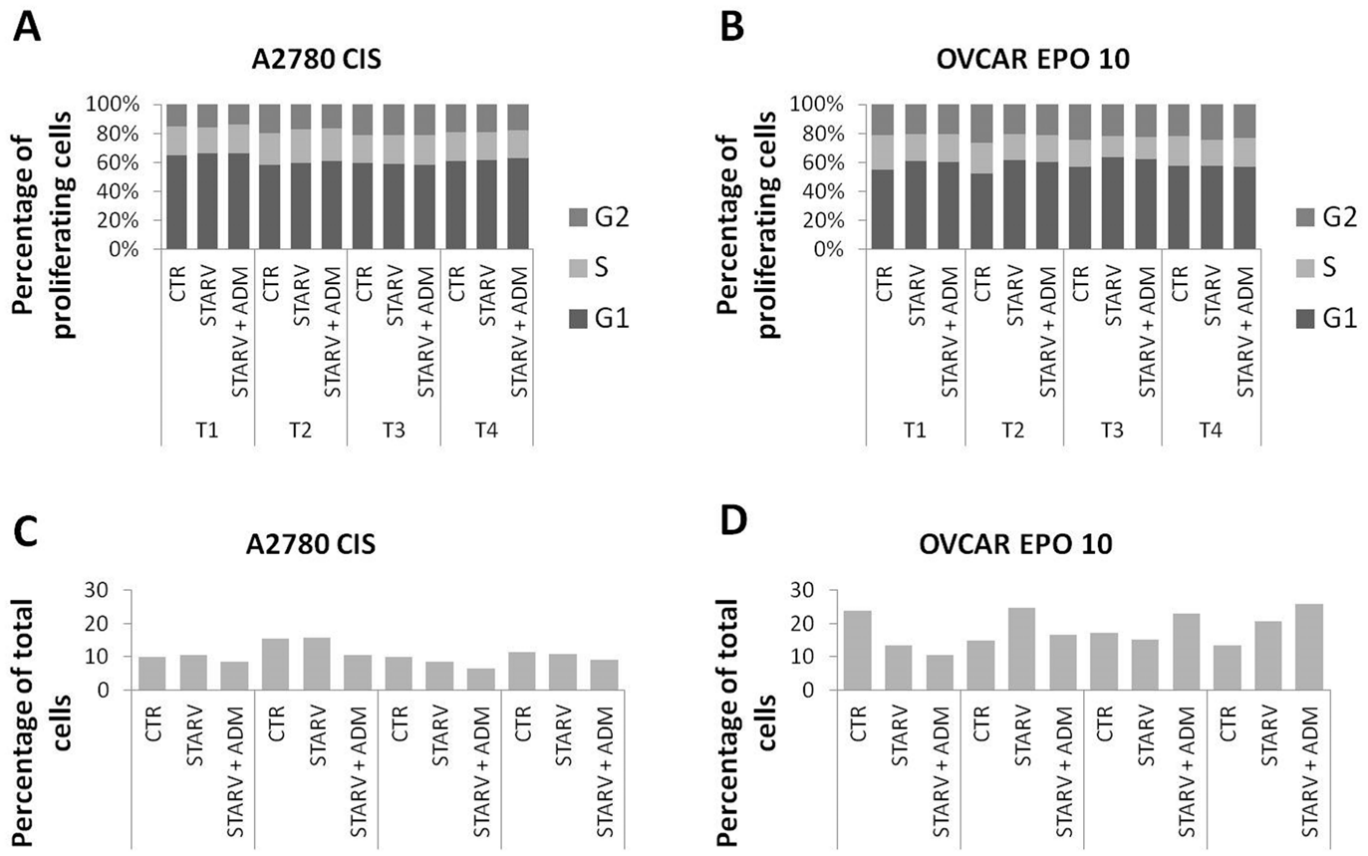
Effect of ADM on cell cycle in A2780CIS and OVCAR-EPO cells. Cells were treated as described in Figure 2. No relevant effects were noticed in these 2 cell lines in terms of modulation of cell cycle and death induced by ADM. This experiment was repeated twice with similar results.

In order to evaluate the effects of ADM on the growth of ovarian cancer cell lines, OVCAR-3, OVCAR-EPO10, and A2780 and its paclitaxel-resistant counterparts TC1 and TC3 cells were treated with recombinant human ADM at 1, 10, and 100 nM concentration for 72 hours. After harvesting, the cells were counted. A modest effect on cell growth was noticed in some cell lines, such as A2780, TC1, and OVCAR-3 cells (Fig. 1). Thereafter, the effect on the cell cycle was analyzed upon supplementation with exogenous ADM and concomitant serum starvation. Cells were seeded in 24-well plates and serum starved for 36 hours with or without addition of ADM 100 nM. After 36 hrs (T1), 5×10^5^ cells from each condition were harvested and DNA analysis was performed. To gauge the recovery of the cells, in the other wells medium was replaced with FBS-containing medium. Cells were harvested after 4 (T2), 8 (T3), and 24 hrs (T4), and again DNA analysis was performed. Percentage of cells in each phase was evaluated and representative data are shown for A2780 and OVCAR-3 cells (Fig. 2A–B) and for their drug-resistant counterparts A2780CIS and OVCAR-EPO (Fig. 3 A–B), which are resistant to cisplatin and patupilone, respectively. A modest increase in the S phase was noticed in the samples treated with ADM. DNA fragmentation, including in the sub-G_0/1_ peak, was also evaluated as an indicator of cell death (Figs. 2 C–D, 3C–D). The effect of ADM on cell death was not detectable. Taken together, these findings suggest that ADM has a modest role as a growth and survival factor in the ovarian cancer cell lines analyzed.

**Figure 4:**
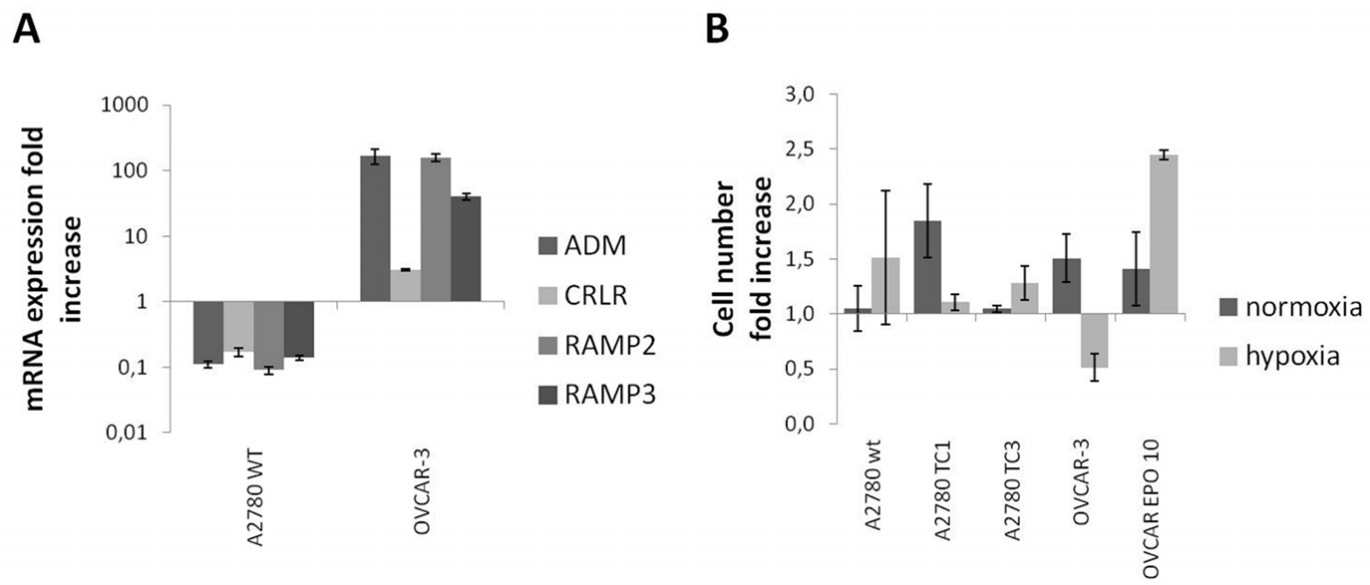
Effect of ADM under hypoxic conditions. A2780 and OVCAR-3 cells were cultured under hypoxic conditions for 72 hrs with and without adding ADM 100 nM. RNA extracted from cell pellets was analyzed by qPCR. In A2780 cells, which are sensitive to hypoxia, ADM and ADM receptors were downregulated in hypoxic conditions. In OVCAR-3 cells, which are less sensitive to hypoxia, ADM and ADM receptors were upregulated (A). A2780, TC1, TC3, OVCAR-3, and OVCAR EPO 10 cells were cultured as described above and counted. Treatment with ADM did not modulate ovarian cancer cell growth in either normal or hypoxic conditions, with the exception that in the OVCAR EPO 10 line there was a 2.4-fold growth increase under hypoxic conditions. Parental OVCAR-3 cells were sensitized to hypoxia by ADM . As compared with the normoxic control, hypoxia reduced the number of cells by about 50%. Bar and error bars refer to mean and SD of 2 independent experiments performed in triplicate.

### ADM is a Selective Survival Factor

The effect of hypoxia was investigated in A2780 and OVCAR-3 cells (Fig. 4A). In A2780 cells, which are sensitive to hypoxia, ADM and ADM receptors were downregulated in hypoxic conditions. In OVCAR-3, which are more resistant to hypoxia, ADM and ADM receptors were upregulated. A2780, TC1, TC3, OVCAR-3, and OVCAR EPO 10 were cultured in hypoxic conditions with or without supplementation of ADM 100 nM (Fig. 4B). Among the analyzed cell lines, we noticed that only for OVCAR EPO 10 there was a 2.4 fold increase in hypoxic conditions in the presence of ADM, a different behavior from the parental cell line OVCAR-3, which ADM seemed to sensitize to hypoxia, since only half the number of cells was observed compared to the number found in the sample exposed to hypoxia without ADM. No relevant changes were detectable in the other models.

**Figure 5:**
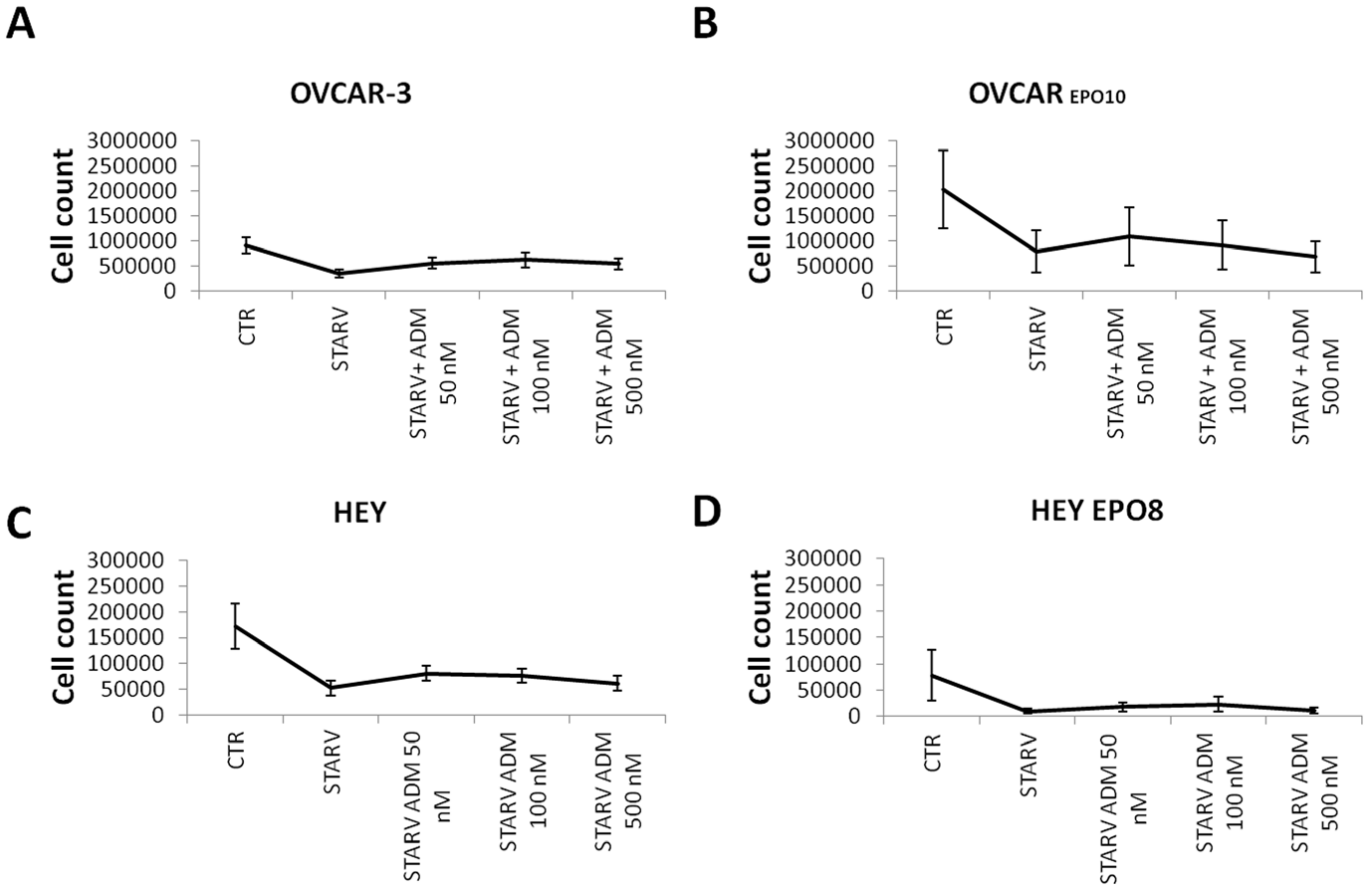
Effect of ADM on cell growth under conditions of serum starvation. OVCAR-3 (A), OVCAR EPO10 (B), HEY (C), and HEY-EPO8 (D) cells were cultured in serum starvation conditions (without FBS) for an appropriate interval (allowing only about 50% of cells to proliferate), with or without addition of ADM 50 nM, 100 nM, and 500 nM. Cell counts are shown as the total number of cells. Remarkably, the presence of ADM did not noticeably affect cell growth. Each datapoint and error bar refers to mean and SD of 2 independent experiments performed in triplicate.

**Figure 6:**
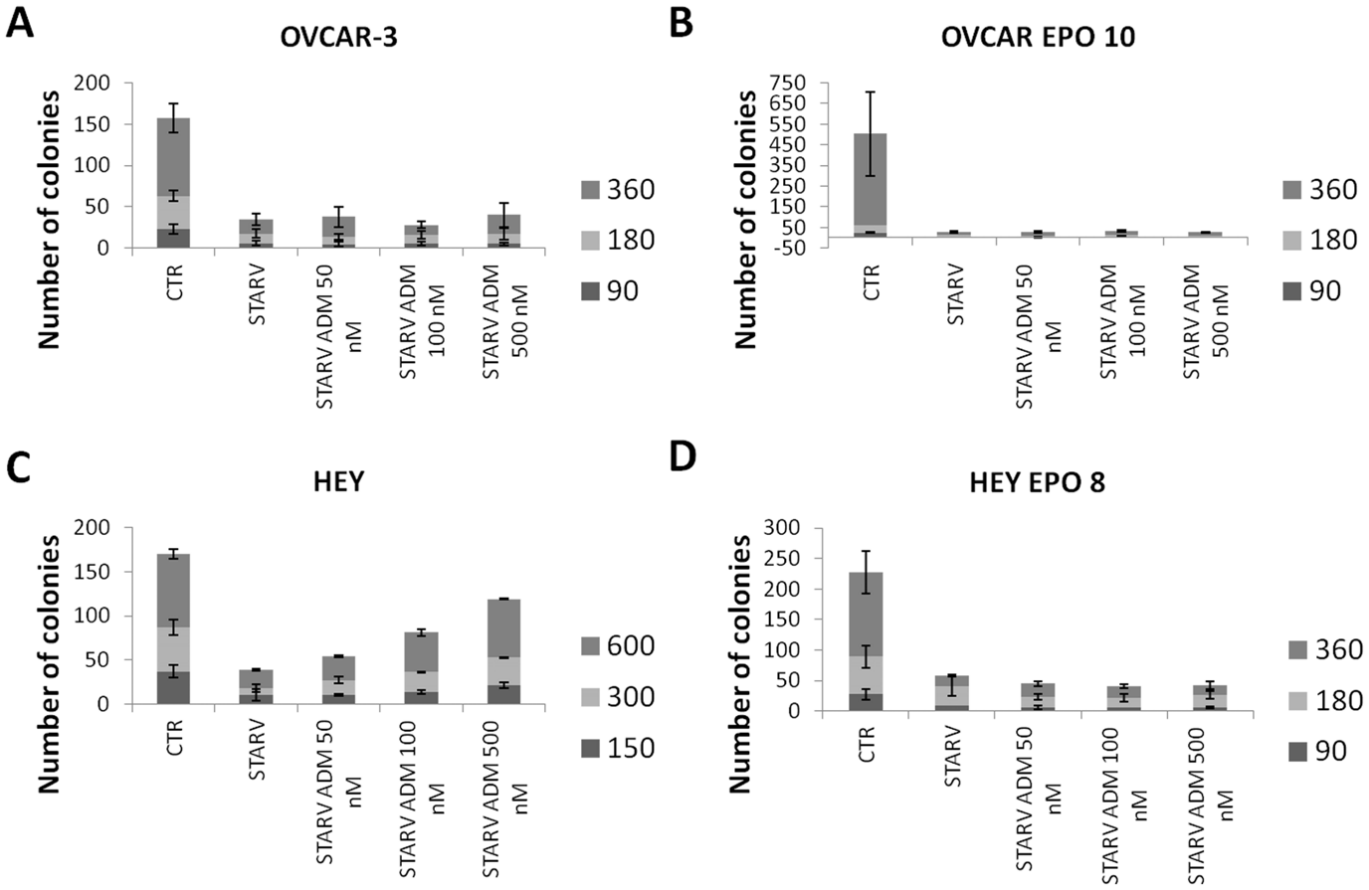
Effect of ADM on clonogenic capacity. OVCAR-3 (A), OVCAR EPO10 (B), HEY (C), and HEY-EPO8 (D) cell suspensions from the serum starvation experiment were diluted and plated on Petri dishes at 3 different cell seedings per dish. The effect of ADM on clonogenic capacity after 14 days is shown as the number of colonies. Only HEY cells showed any consistent effect of protection from serum starvation after ADM treatment in this assay, while their epothilone-resistant counterparts exhibited the opposite effect. Bar and error bars refer to mean and SD of 2 independent experiments performed in triplicate.

In order to study the effect of ADM treatments in a different stress model, cells were seeded in 6-well plates and serum starved. An incubation period was established after preliminary assays (36 hours for HEY and OVCAR EPO10, 48 hours for HEY EPO8, and 60 hours for OVCAR-3). Serum starvation conditions alone, without ADM, induced at least 50% of growth inhibition. ADM was added at 3 different concentrations (50 nM, 100 nM, and 500 nM). Data from the proliferation assays in serum starvation conditions are shown in Figure 5A–D. Cells from each assay were then plated in Petri dishes at 3 different cell seeding densities per dish (90, 180, and 360 cells were seeded in each dish for HEY EPO 8, OVCAR-3, and OVCAR EPO10; 150, 300, and 600 cells/dish for HEY) and colonies were counted after 14 days. As with hypoxia, serum starvation was effective in reducing the number of cells in short-term 3-day growth assays. A modest, not statistically significant increase was noted when ADM was added at the lowest concentration. In order to assess the presence of a protective effect of ADM in the clonogenic compartment, after exposure to serum starvation, cells were plated at limiting dilutions and the colonies counted after 10–14 days (Fig. 6A–D). In this assay, only in HEY cells a consistently protective effect of serum starvation was noted with ADM treatment, while in the other cell types no changes were recorded. Taken together, these findings demonstrated that only in some cell lines was ADM capable of mediating growth or survival factors.

**Figure 7:**
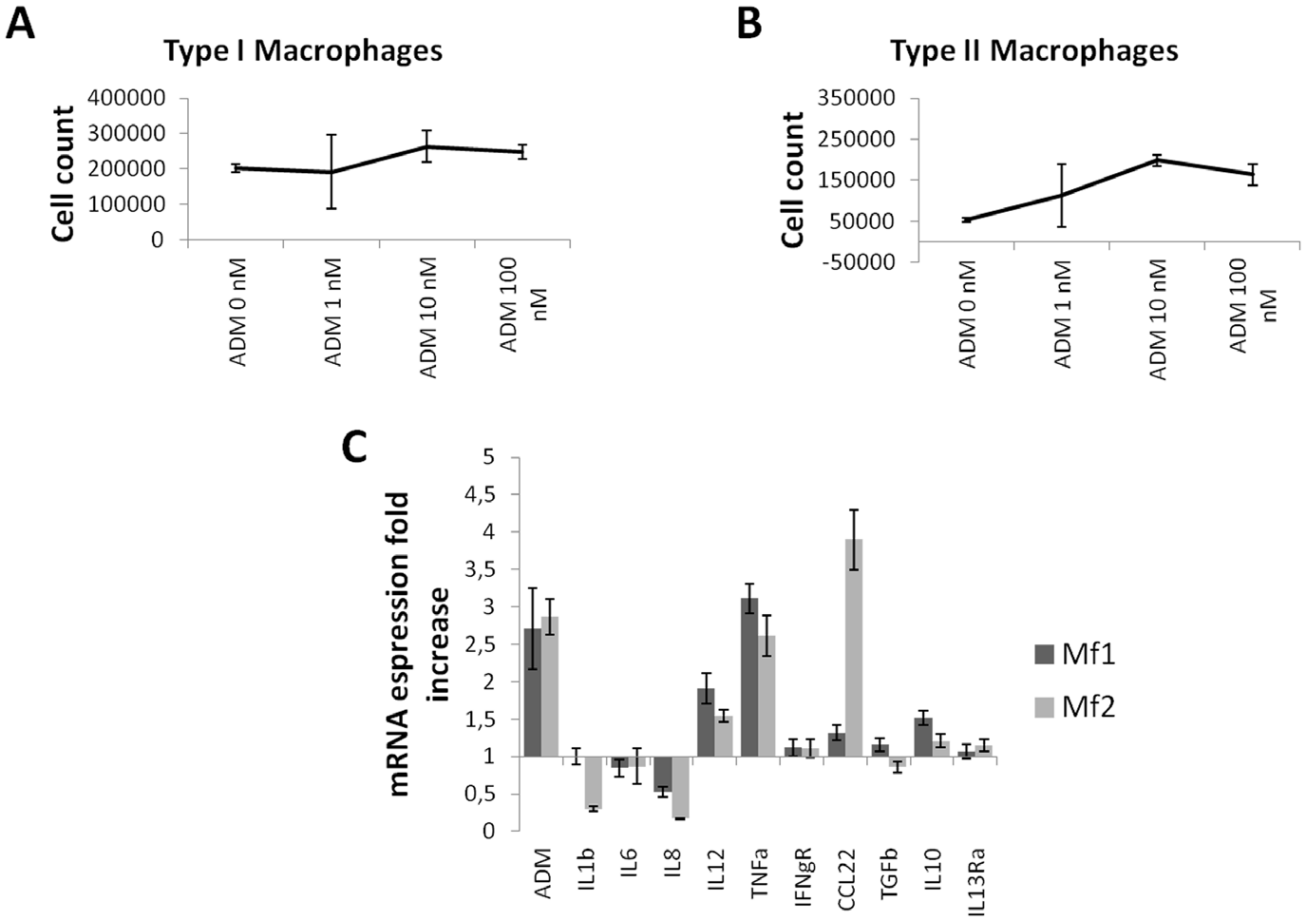
Effect of ADM on macrophage proliferation and differentiation. Type 1 (A) and type 2 (B) macrophages were treated with ADM 1 nM, 10 nM, and 100 nM for 72 hours and counted. Cell counts are shown as the absolute number of cells. Type 2 cells cultured in the presence of ADM reached a number up to 3.8-fold higher than the count for untreated cells, while a lesser increase can be observed in type 1 macrophages (1.3-fold higher with ADM 10 nM). Expression of endogenous ADM, IL1β, IL6, IL8, IL12, TNFα, IFNγR, CCL22, TGFβ, IL10, and IL13Rα was evaluated by qPCR (C) after treatment of type 1 and type 2 macrophages with rhADM 100 nM for 24 hours. In both types of cells, endogenous ADM, IL-12, and TNF-α are upregulated. IL1β and IL8 were downregulated while CCL22 is upregulated in type 2 macrophages versus type 1. No other relevant differences in the expression of the other cytokines, chemokines, and receptors were noticed. Bar and error bars refer to mean and SD of 2 independent experiments performed in triplicate.

**Figure 8:**
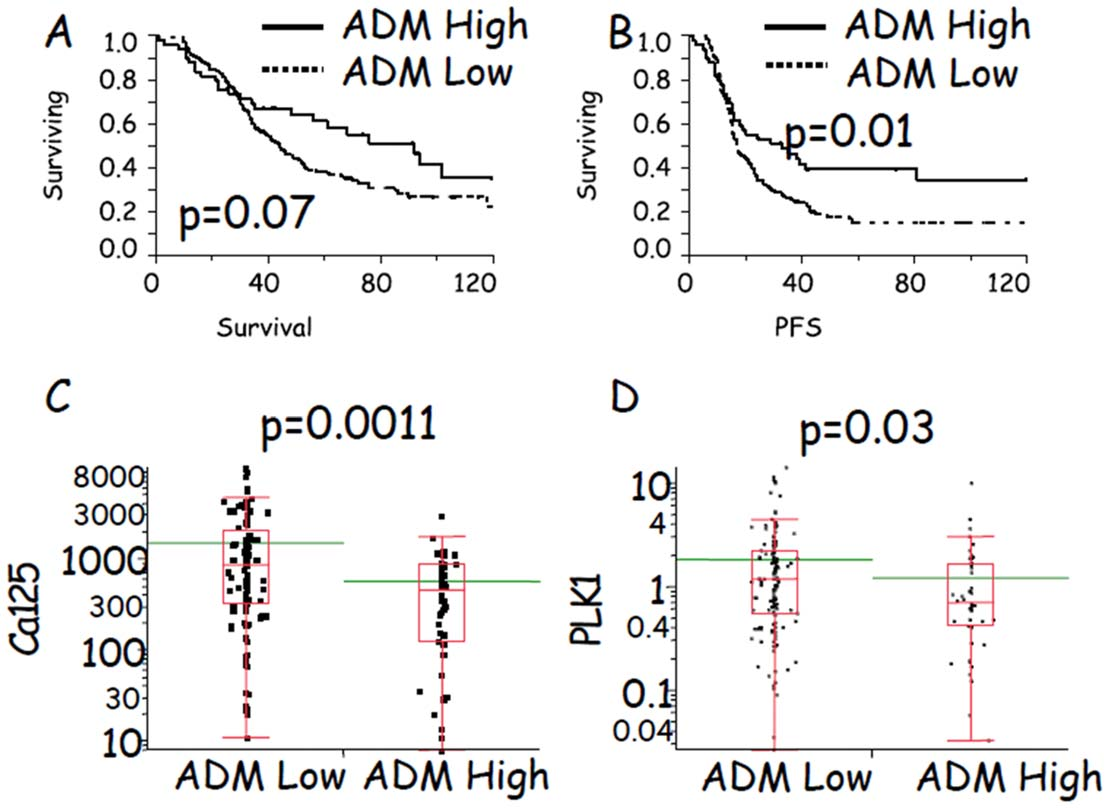
Evaluation of ADM as a prognostic factor in ovarian cancer patients. Kaplan-Meier analysis of 220 ovarian cancer patients (A) revealed that high expression levels of ADM were related to a positive outcome in terms of OS, although the difference did not achieve statistical significance (*p* = 0.07). The same analysis, with a final endpoint of PFS, revealed the same trend (B), ie, patients with lower expression of ADM relapsed earlier than those with higher levels (*p* = 0.01). Multivariate analysis showed that age, clinical stage, histotype, and grading were not significantly related to ADM expression, while there was a strong significant inverse association (*p*<0.001) between levels of Ca125 and ADM expression (C). Also, levels of the PLK1 gene, which is related to the number of cells in M phase, were significantly reduced in the group with low ADM expression (D).

### ADM Affects Monocyte/Macrophage Proliferation and Differentiation

Because macrophages are central to the production of ADM in the stroma, type 1 (M1) and type 2 (M2) macrophages were treated with 3 different concentrations of ADM (1 nM, 10 nM, and 100 nM) for 72 hours. Cells were harvested by trypsinization and counted. M2 cultured in the presence of ADM reached a count up to 3.8-fold higher than the count for untreated cells, while a lower increase was observed in M1 (1.3 with ADM 10 nM), indicating that somehow ADM was able to modulate macrophage differentiation and proliferation (Fig. 7A). As confirmation of the capacity of ADM to modulate macrophage differentiation, M1 and M2 were treated with ADM 100 nM for 24 hours. Gene expression of ADM, IL1β, IL6, IL8, IL12, TNFα, IFNR, CCL22, TGFβ, IL10, and IL13R was assessed through qPCR. Upregulation of endogenous ADM was observed in M1 and M2, as well as upregulation of IL-12 and TNF-α (Fig. 7B), indicating that ADM was able to regulate its expression in macrophages in an autocrine manner. The only differences noted in cytokine/chemokine expression in response to ADM were downregulation of IL1β and IL8 and upregulation of CCL22 in M2 vs. M1.

**Table 1:** Clinical Features of the analyzed setting of ovarian cancer patients.

Characteristics	No. pts (%)
**All cases**	220
**Age, yrs**	
Median	58.5
**FIGO Stage**	
I–II	29 (13.1)
III	163 (74.1)
IV	28 (12.8)
**Histotype**	
Papillary-serous	161 (73.2)
Mucinous	5 (2.2)
Endometrioid	28 (12.7)
Clear Cell	13 (5.9)
Undifferentiated	10 (4.5)
Adenocarcinoma	3 (1.4)
(not specified)	
**Ca 125**	
Median	625 U/mL
(range)	(12.5–10000)
**Status**	
Dead	139 (63.1)
Alive	81 (36.9)
Median follow up	71 months
(Alive)	

**Table 2:** Univariate and multivariate Coxs proportional hazard ratio in 220 ovarian cancer patients.

	*n* (%)	HR	95% CI	*P*-value
*Overall Survival (Univariate analysis)*				
Age	220/220 (100)	2.17	0.99–4.77	0.0528
Ca125	179/220 (81)	1.36	0.45–3.48	0.5562
Stage III/I–II*	192/192 (100)	2.92	1.56–6.21	<0.0001
Stage IV/I–II*	57/57 (100)	6.05	2.88–13.85	<0.0001
Endometrioid**	189/189 (100)	0.37	0.17–0.68	0.0109
ADM	195/220 (89)	0.21	0.01–1.27	0.0079
PLK1	205/220 (93)	1.01	0.93–1.09	0.6456
*Progression Free Survival (Univariate analysis* )				
Age	220/220 (100)	1.26	0.63–2.51	0.5046
Ca125	179/220 (81)	3.42	1.39–7.56	0.0086
Stage III/I–II*	192/192 (100)	5.25	2.74–11.70	<0.0001
Stage IV/I–II	57/57 (100)	8.56	4.02–20.32	<0.0001
Endometrioid**	189/189 (100)	0.30	0.16–0.52	<0.0001
ADM	195/220 (89)	0.18	0.04–0.78	0.0027
PLK1	205/220 (93)	0.97	0.33–2.42	0.9605
*Overall Survival (Multivariate analysis)*				
Stage III/I–II*	192/192 (100)	2.12	1.03–4.95	0.0395
Stage IV/I–II*	57/57 (100)	3.86	1.66–9.77	0.0014
Endometrioid**	189/189 (100)	0.42	0.17–0.88	0.0195
ADM	195/220 (89)	0.18	0.02–1.09	0.0141
*Progression Free Survival (Multivariate analysis)*				
Ca125	179/220 (81)	1.77	0.61–4.51	0.2718
Stage III/I–II*	192/192 (100)	6.76	2.40–28.3	<0.0001
Stage IV/I–II*	57/57 (100)	9.87	3.19–43.39	<0.0001
Endometrioid**	189/189 (100)	0.61	0.28–1.19	0.1610
ADM	195/220 (89)	0.56	0.04–1.78	0.0268

CI = confidence interval; HR = hazards ratio;

* HR was calculated Stage III vs. Stage I-II;**HR was calculated endometrioid vs. serous papillary Histotype; in both cases all the other interactions were not significant.

### High Expression Levels of ADM was a Positive Prognostic Factor in Ovarian Cancer Patients

In order to gain insight into the role of ADM in ovarian cancer, a clinical cohort of 220 ovarian cancer patients was analyzed retrospectively using a nanofluidic genetic analyzer and a 48.48 chip. Ca125 analysis and patient specimens were collected at first surgery, prior any treatment. GAPDH served as housekeeping gene and results were normalized for the expression levels detected in OVCAR-3 cells. The description of patients enrolled in this study is summarized in Table 1. A preliminary outcome analysis was performed using the Kaplan-Meier method. High expression levels of ADM seem to be linked to better survival, although the difference did not achieve statistical significance (*p* = 0.07) (Fig. 8A). The same analysis, with its final endpoint progression-free survival (PFS), revealed the same trend (Fig. 8B), with patients having lower expression of ADM relapsing earlier than those with higher levels (*p* = 0.01). In order to gain insight into these findings, we employed multivariate analysis to evaluate those clinical parameters that were related to ADM expression. Spearmans correlation was used to assess the relationships among ADM, Ca125, and age taken as continuous variables. No significant correlations were noted between ADM and age (*ρ* = −0.008, *p* = 0.91) and Ca125 and age *(ρ* = −0.139, *p* = 0.17), while a significant inverse correlation was found between Ca125 and ADM (*ρ* = −0.23, *p* = 0.003). ADM values were also analyzed by histological type and stage of disease using the Kruskal-Wallis test. ADM expression did not change when compared between histotypes (χ^2^ = 5.14, *p* = 0.39) or clinical stage (χ^2^ = 3.94, *p* = 0.14). When patients were stratified in high and low expression levels, there was again a significant inverse association (χ^2^ = 10.7, *p*<0.01) between levels of Ca125 and ADM expression (Fig. 8C). These data suggest that tumors with lower expression of ADM have a greater mass than those with higher ADM expression. In keeping with this hypothesis, the levels of PLK1–a factor related to the number of cells in M phase–were also significantly reduced (χ^2^ = 4.6, *p* = 0.03) in the group with low ADM expression (Fig. 8D). These findings suggest that high expression of ADM exerts growth-suppressive effect on ovarian cancer cells and is linked to positive outcome. To assess this hypothesis, Cox univariate analysis, which took into account clinical stage, histotype, age, Ca125, ADM, and PLK1 (the latter 4 parameters were taken as continuous variables) was performed using both OS and PFS as variables for outcome (Table 2). Variables capable to predict the outcome in univariate analysis were selected to perform Cox multivariate analysis. As previously reported [12], clinical stage was the most important variable to predict the risk of death in ovarian cancer patients. This fact was evident in both OS and PFS analysis in univariate and multivariate analysis. The histotype endometrioid was a characteristic of those patients with a better OS and PFS in univariate analysis, an effect maintained in multivariate analysis for OS. High expression levels of ADM were related to better OS and PFS. This effect, although modest in terms of risk, was maintained in both univariate and multivariate analysis, supporting the hypothesis that high expression levels of ADM are predictive of positive outcome in ovarian cancer patients.

**Table 3:** List of primer oligonucleotides designed for qPCR analysis.

GAPDH	Forward:	5′-CCTGACCTGCCGTCTAGAAA-3′
	Reverse:	5′-CTCAGTGTAGCCCAGGATGC-3′
ADM	Forward:	5′-TGCCCAGACCCTTATTCGG-3′
	Reverse:	5′-AGTTGTTCATGCTCTGGCGG-3′
CRLR	Forward:	5′-ACCCCTTCAACAAGCAGAA-3′
	Reverse:	5′-TCAGTTCCTGCTGCAACATC-3′
RAMP2	Forward:	5′-GACGGTGAAGAACTATGAGACAGC-3′
	Reverse:	5′-GCTATAAGGCCTGCTAATCATGG-3′
RAMP3	Forward:	5′-CACAGGCAGTTCTTCTCCAACT-3′
	Reverse:	5′-GACAGTCAGAACGACGGGTATAA-3′
IL1β	Forward:	5′-AAGTGGTGTTCTCCATGTCCTT-3′
	Reverse:	5′-CAGCTGTAGAGTGGGCTTATCAT-3′
IL6	Forward:	5′-CCAATCTGGATTCAATGAGGAG-3′
	Reverse:	5′-GCTCTGGCTTGTTCCTCACTAC-3′
IL8	Forward:	5′-ATTTCTGCAGCTCTGTGTGAAG-3′
	Reverse:	5′-GGTCCACTCTCAATCACTCTCAG-3′
IL12p40	Forward:	5′-TCGGCAGGTGGAGGTCAGC-3′
	Reverse:	5′-CGCAGAATGTCAGGGGAAGTAGG-3′
TNFα	Forward:	5′-AGAGGGCCTGTACCTCATCTACT-3′
	Reverse:	5′-GAGGTTGACCTTGGTCTGGTAG-3′
IFNγR	Forward:	5′-CATCACGTCATACCAGCCATTT-3′
	Reverse:	5′-CTGGATTGTCTTCGGTATGCAT-3′
CCL22	Forward:	5′-GTGATTACGTCCGTTACCGTCT-3′
	Reverse:	5′-TCTCCTTATCCCTGAAGGTTAGC-3′
TGFβ	Forward:	5′-GACACCAACTATTGCTTCAG-3′
	Reverse:	5′-CAGGCTCCAAATGTAGGG-3′
IL10	Forward:	5′-CCTTCAGCAGAGTGAAGACTTTC-3′
	Reverse:	5′-TAACCCTTAAAGTCCTCCAGCA-3′
IL13Rα	Forward:	5′-CTCCACCAGTCATTTTTCAG-3′
	Reverse:	5′-ATTATCCTCTGCTCCTCCAG-3′

## Discussion

Strong preclinical and translational studies support the hypothesis that stromal components play a pivotal role in driving an aggressive tumor phenotype. In this context, ADM is a ubiquitous regulatory peptide with several biologic functions, such as vascular action, growth-stimulating effects, and immune-modulating activity. ADM is highly expressed in a variety of malignancies such as glioblastoma [13], clear-cell renal carcinoma [14] and pancreatic cancer [15]. In all these diseases ADM has been considered a biomarker of poor outcome. This characteristic of ADM has been ascribed mainly to its mitogenic properties and to its ability to function as a survival factor induced by hypoxia. Along with its direct effects on cancer cells’ survival, an additional factor in tumor aggressiveness consists in the capacity of ADM to stimulate angiogenesis inside the tumor. This mechanism seems particularly relevant in clear-cell renal carcinoma, a disease characterized by intensified vascularization inside the tumor [16]. Few reports are available about whether this phenomenon occurs in ovarian cancer and about the precise function of ADM in this disease. In a small clinical setting of 60 ovarian cancer patients, using nonquantitative PCR analysis, in 2000 Hata and colleagues reported that ADM was a factor predictive of poor outcome [17]. Apart from this report, no other translational studies addressed the role of ADM in ovarian cancer. The effects of ADM on ovarian cancer cells in in vitro cultures is controversial. Giacalone and colleagues reported that ADM was unable to stimulate cell growth in BG-1, PEO4, and PEO14 ovarian cancer cells [18]. Conversely, a recent study reported that silencing of the ADM gene in HO8910 ovarian cancer cells was accompanied by growth inhibition and chemosensitization through downregulation of ERK and bcl-2 expression [19]. These findings prompted us to investigate whether ADM is a mitogenic survival factor in a panel of cancer cells exhibiting various degrees of drug resistance. In vitro analysis demonstrated that ADM showed minimal activity as a growth factor, an effect that varied according to cell line, with a modest increase in cells in the S phase of the cell cycle. The activity of ADM as a survival factor was sharply differentiated according to cell line. In Hey cells, after serum starvation there was a consistent increase in the number of cells capable of surviving and forming colonies, while in other cellular models such an effect was barely detectable. Extreme variability was also observed in terms of the response to hypoxia. In this response we noted a reduction in the expression of ADM and its receptors in cells sensitive to hypoxia, such as A2780, while in OVCAR-3 the opposite trends were observed. By combining the results reported in the literature and our own findings we concluded that ADM exerts a modest effect as a growth and survival factor in ovarian cancer cells, with extreme variability depending upon the cell model analyzed.

A recent report on melanoma suggested that the major source of ADM is macrophages [6]. In order to verify such a hypothesis, we looked at the ability of ADM to stimulate the growth of polarized M1 and M2 macrophages. ADM was able to regulate their production in an autocrine way and selectively enhanced the proliferation of M2, which has a low IL-12/high IL-10 phenotype, and which exhibited impaired expression of reactive nitrogen intermediates, lower antigen presentation and tumoricidal capacity, and high expression of angiogenic factors such as VEGF, EGF, and semaphorin 4D [20]. High expression of M2 is therefore generally associated with an immune environment that renders cancer more aggressive.

Despite these in vitro findings, our translational study revealed, surprisingly, that patients with higher expression of the ADM gene tended to have longer PFS and a better outcome. We noted, in particular, an inverse correlation between expression levels of ADM and Ca125 measured at the time of first surgery. It is commonly accepted that Ca125 is a marker for tumor volume. In ovarian cancer that responds to treatment, a reduction in the expression of Ca125 is notable. Tumors characterized by low levels of ADM also exhibit higher expression of PLK1, a marker of mitotic activity. Therefore it seems likely that, in patients, ADM exerts, directly or indirectly, a growth-suppressive effect, with a reduction of cells in the M phase of the cell cycle suggested by the expression of PLK1. In a large number of studies, the major effect seen for in vivo ADM-stimulated cells was an elevation in cAMP (reviewed in [8]). Agents that increase cAMP in cancer cells have often been associated with inhibition of cell proliferation [21], and several drugs capable of mimicking cAMP are in clinical development as anticancer agents [22]. Along with a direct effect on cancer cells through cAMP, it is also possible that patients with higher levels of ADM exhibit tumors with better vascularization, as demonstrated in clear-cell renal carcinoma [16]. Since ovarian cancer is a chemosensitive disease in most patients [12] we can speculate that the tissue concentration of chemotherapeutics is increased when levels of ADM are elevated, leading to better control of the disease and a longer disease-free interval.

In summary, this study revealed that although ADM can behave as a growth/survival factor in vitro, and as an agent capable of selectively stimulating M2 polarization, these properties were not confirmed in patients. Therefore this study does not support the use of ADM antagonists as a tool for improving the management of ovarian cancer patients.

## Materials and Methods

### Cell Lines and Reagents

A2780 and OVCAR-3 cells lines were obtained from ATCC. A2780 is a human ovarian cancer cell line derived from the tumor tissue of an untreated patient. A2780 TC1 and A2780 TC3 are Paclitaxel-resistant cell lines obtained from A2780 after prolonged treatment with cyclosporine and Paclitaxel (kindly provided by Indena). OVCAR EPO10 and HEY EPO8 are Epothilone B (EpoB)-resistant cell lines obtained from OVCAR-3 and HEY respectively, after prolonged treatment with EpoB. All of these cell lines have been described previously [23]. Cell lines were cultured under standard conditions (37°C, 5% CO_2_) in RPMI 1640 with glutamine supplemented with 10% fetal bovine serum (FBS), 0.1 mM MEM nonessential amino acids and 0.1 mg/ml kanamycin. All reagents were purchased from Sigma-Aldrich if other vendors were not specified. Experiments in hypoxia were performed as previously described [24].

### Preparation of Macrophage Primary Cultures

Lymphomonocytes were isolated from the buffy coat of a healthy donor by density centrifugation gradient using Histopaque-1077 (Sigma-Aldrich) and resuspended in RPMI 1640 supplemented with 10% human AB serum (Sigma-Aldrich), 100 IU/ml penicillin and 100 µg/ml streptomycin (GIBCO). About 10^7^ lymphomonocytes per well were seeded in 6-well primary culture plates (BD Falcon) and incubated at 37°C 5% CO_2_ for 2 hours to allow monocytes (10% to 30%) to adhere to the bottom of the wells. Fresh culture medium containing either 50 ng/ml recombinant human GM-CSF or 50 ng/ml recombinant human M-CSF (R&D Systems) was added to allow monocytes to differentiate toward type 1 and type 2 macrophages, respectively. Type 1 and type 2 macrophages were treated with recombinant human ADM (Sigma-Aldrich), then washed twice with pre-warmed PBS. Cells were collected in sterile tubes and centrifuged for RNA extraction or counted for cell proliferation analysis.

### RNA Extraction and Reverse-Transcription Polymerase Chain Reaction (PCR)

Total RNA was extracted from 1–3×10^6^ cells using the BioRobot EZ1 system (Qiagen) following the manufacturer’s instructions. For reverse transcription, the iScript^TM^ cDNA synthesis kit (Biorad) was used. qPCR analysis was performed using the iQ™ SYBR^®^ Green Supermix and the DNA Engine Opticon 2 detection system (Biorad). Glyceraldehyde 3-phosphate dehydrogenase (GAPDH) was used as housekeeping gene. The sequences of primer oligonucleotides are listed in Table 3.

### Cell Cycle Analysis

Ovarian cancer cells were seeded in 24-well plates at 1–5×10^4^ cells/well. In some conditions cells were starved (deprived of fetal bovine serum, or FBS) in the presence of ADM 100 nM. Controls were represented by cells starved without adding ADM (negative control) and by cells cultured in complete medium (positive control). After 36 hrs (T1), 5×10^5^ cells from each condition were harvested by trypsinization, collected in flow cytometry tubes, and labelled as previously described [25]. Each sample was acquired using the Cytomics™ Flow Cytometer (Beckman Coulter).

### Cell Proliferation Assays

Ovarian cancer cells in culture medium were seeded in 6-well plates at predetermined optimal cell densities (HEY: 6×10^4^/well; A2780, A2780 TC1, A2780 TC3, and OVCAR EPO10: 8×10^4^/well; OVCAR-3 and HEY EPO8: 10^5^/well). Recombinant human ADM was added to the medium after adhesion of cells to the bottom of the wells. After 72 hours, floating cells in the supernatant were harvested. Adherent cells were treated with trypsin-EDTA and collected, together with their corresponding supernatants. Total cell counts were performed using a hemocytometer (Burker chamber, Nalgene). Hypoxic conditions were performed by incubating cells in a Billrop chamber.

In some experiments, HEY, HEY EPO8, OVCAR, and OVCAR EPO10 cells were cultured under starvation conditions (without FBS) for predetermined times, with or without adding ADM. The positive control was constituted by cells cultured in complete medium.

Type 1 and type 2 macrophages were treated with rhADM for 72 hours, then harvested after treatment with trypsin-EDTA and counted in a Burker chamber.

### Clonogenic Assays

After counting OVCAR-3, OVCAR EPO10, HEY, and HEY EPO8 cells from serum starvation experiments, each cell suspension was diluted in FBS-containing medium to very low cell densities. For OVCAR-3, OVCAR EPO10, and HEY EPO8, each solution was diluted to 36, 18, and 9 cells/ml, then 10-ml aliquots of each cell suspension were placed in Petri dishes so that wells were populated with 360, 180 and 90 cells per dish, respectively. For HEY cells, each solution was diluted to 60, 30, and 15 cells/ml so that cell densities in 10-ml suspensions each well were 600, 300, and 150 cells per dish. Petri dishes were incubated at 37°C 5% CO_2_ until colonies could be seen (10–14 days). Colonies were fixed and stained with a solution of 1% methylene blue (Sigma) in 70% ethanol, then viewed and counted using an Image Station 4000 M Digital Imaging System (Kodak) and molecular imaging software (Carestream).

### Nanofluidic Analysis of Gene Expression

FFPE samples were obtained from ovarian cancer sections preserved between the years 2000 and 2008 following the approved Danbury Hospital Internal Review Board protocol. All patients provided appropriate written consent to participate to the study. FFPE samples were cut to 10 µm thickness and 2 tissue slices were placed in a 1.5 ml tube. One milliliter of xylene was added for deparaffinization, followed by mixing twice with a high-speed vortex for 3 min at room temperature. Total RNA was then automatically extracted with the QIAcube using the Qiagen miRNeasy FFPE kit (Valencia, CA) following the manufacturer’s protocols. RNA from the cell line A2780 was automatically extracted with the QIAcube using the Qiagen miRNeasy kit (Valencia, CA) following the manufacturer’s protocols. RNA quantity and the quality were assessed using the Agilent 2100 Bioanalyzer (Agilent Technologies, Santa Clara, CA). Analysis was carried out using the 48.48 dynamic array (Fluidigm Corporation, CA, USA) and a Biomark platform, following the manufacturers’ protocols.

### Statistical Analysis

Overall survival (OS) and progression free survival (PFS) were calculated from the date of diagnosis to the date of progression/death or date last seen. Medians and life tables were computed using the product-limit estimate by the Kaplan-Meier method and the Wilcoxon test was employed only to assess statistical significance. Multivariate analysis assessed the clinical role of ADM expression matched with other clinical variables (age, stage, grading, histotype, Ca125 values) using the Cox proportional hazards model and nonparametric testing with the Kruskal Wallis test. Statistical analysis was carried out using JMP9 (SAS).
